# Pathological molecular mechanism of symptomatic late-onset Fuchs endothelial corneal dystrophy by bioinformatic analysis

**DOI:** 10.1371/journal.pone.0197750

**Published:** 2018-05-22

**Authors:** Zekai Cui, Qiaolang Zeng, Yonglong Guo, Shiwei Liu, Peiyuan Wang, Mengyuan Xie, Jiansu Chen

**Affiliations:** 1 Key Laboratory for Regenerative Medicine, Ministry of Education, Jinan University, Guangzhou, Guangdong, P.R. China; 2 The Department of Ophthalmology, the First Clinical Medical College, Jinan University, Guangzhou, Guangdong, P.R. China; 3 Key Laboratory of Optoelectronic Information and Sensing Technologies of Guangdong Higher Educational Institutes, Jinan University, Guangzhou, Guangdong, P.R. China; 4 Institute of Ophthalmology, Medical College, Jinan University, Guangzhou, Guangdong, P.R. China; University of Texas MD Anderson Cancer Center, UNITED STATES

## Abstract

Fuchs endothelial corneal dystrophy (FECD) is a degenerative disease characterized by corneal endothelial decompensation. FECD causes corneal stromal and epithelial edema and progressively develops into bullous keratopathy, which can eventually lead to blindness. However, the exact pathogenesis is unknown. In this study, we performed an in-depth bioinformatic analysis of the dataset GSE74123 to determine the differentially expressed genes (DEGs) of symptomatic late-onset FECD compared with a normal control. Gene ontology (GO) terms and Kyoto Encyclopedia of Genes and Genomes (KEGG) pathways analysis were used to analyze the pathological molecular mechanism of FECD. We found that cell senescence, reactive oxygen species (ROS), the extracellular matrix (ECM), epithelial-mesenchymal transition (EMT) and immune response-related genes play an important role in the pathological development of symptomatic late-onset FECD. In addition, we revealed that down-regulated IL-6, enhanced NF-κB activity and a suite of orchestrated chemokine responses induce fibrocyte differentiation from monocyte to dendritic cell maturation. PI3K plays a key role in the molecular mechanism of symptomatic late-onset FECD. This study enhances our understanding of the molecular mechanism of FECD pathogenesis and will improve the diagnostics and therapy of FECD patients in the future.

## Introduction

Fuchs endothelial corneal dystrophy (FECD), also known as cornea guttata, was originally reported and described by Fuchs in 1910[1]. FECD is a degenerative disease characterized by corneal endothelial decompensation. The characteristic features of FECD are the formation of corneal guttae, local thickening of the corneal Descemet's membrane (DM), a decline in corneal endothelial cell density and ion transport function[2]. The number of corneal endothelial cells (CECs) decreases, causing corneal endothelial dysfunction. Corneal stromal and epithelial edema progressively develop into bullous keratopathy, which can eventually lead to blindness[3]. Corneal transplantation is currently the only way to save a patient's vision[4].

According to the age of onset, FECD can be divided into two subtypes: early-onset (approximately 30 years old) and late-onset (approximately 50 years old). The incidence of late-onset FECD is higher. In America, the prevalence is approximately 4% of the population over the age of 40[5]. There are a variety of criteria for the clinical stage of FECD; the most commonly used system describes the disease progression in four stages[6]. Stage 1 is the corneal guttae period. Corneal guttae protruding from the DM are visible under corneal biomicroscopy and are typical signs of FECD. In Stage 2, as the disease progresses, the number of corneal guttae gradually increase. They gather, fuse with each other and extend to the periphery. Corneal guttae grow along the DM, and the density of corneal endothelial cell decreases. The cells’ hexagonal structure is damaged. The function of the corneal endothelial cell biological pump is impaired and corneal stromal edema increases. In Stage 3, stromal edema is exacerbated, and then epithelial and subepithelial bullae appear[6]. Bullae rupture can cause pain, photophobia, tearing and other symptoms[7, 8], and places a patient at a higher risk of infection. Stage 4, long-term chronic edema leads to the appearance of subepithelial connective tissue with reduced corneal transparency[6]. Corneal neovascularization and corneal scarring may occur with long-term corneal edema, resulting in a further decrease in visual acuity. FECD etiology factors include corneal endothelial cell apoptosis[9], sex hormones[10], inflammation[11], and aqueous humor flow[12] and composition[13], but the exact pathogenesis is unknown.

CECs are in cell cycle arrest and hardly proliferate *in vivo*. Therefore, corneal endothelial damage and losses in FECD are permanent and irreversible. CEC morphology changes are mainly reflected in changes in cell shape and size. Corneal confocal microscopy showed that CECs were pleomorphic, and that cell volume increased. The dark black area caused by corneal guttae was observed in the abnormal CECs[14]. CECs in symptomatic late-onset FECD are reduced in number and appear attenuated, causing progressive stromal edema.

Genetic analysis shows that FECD is associated with some gene mutations. Mutations in the *COL8A2* gene occur in some cases of early-onset FECD[15]. COL8A2 is an extracellular matrix protein that is the main component of DM[16]. The more common delayed-onset FECD usually occurs after age 40 and may be familial. Mutations in the *ZEB1*, *SLC4A11*, *TCF4*, *LOXHD1* and *AGBL1* genes result in late-onset disease in a minority of familial and/or unrelated cases[17–21].

De Roo et al. reported that circulating fibrocytes and their dendritic derivatives are a new aspect of FECD. They used CEC monolayers with DM collected from patients with symptomatic late-onset FECD (clinical stages II to IV) during endothelial keratoplasty (CEC transplantation). They performed RNA microarray expression analysis (MEA) and Ingenuity Pathway Analysis (IPA), listed differentially expressed genes and plotted gene network comprising MHC class II molecules. The data suggest that both processes epithelial-mesenchymal transition (EMT) and circulating fibrocytes play a role in FECD. In this study, we used a bioinformatic approach which was different from original research, performed an in-depth bioinformatic analysis of the dataset GSE74123 to determine the differentially expressed genes (DEGs) of FECD compared with the normal control. First, the pathological molecular mechanism of FECD is obtained from Gene ontology (GO) terms and Kyoto Encyclopedia of Genes and Genomes (KEGG) pathway analysis. Then we try to explore the molecular mechanisms for the EMT and fibrocyte differentiation (monocyte/macrophage-dendritic cell maturation) in the corneal endothelial layer of symptomatic late-onset FECD. This study enhances our understanding of the molecular mechanism of FECD pathogenesis and will improve diagnostics and therapy for FECD patients in the future.

## Materials and methods

### Microarray data of FECD and normal control

The data set GSE74123 provided by De Roo et al. was downloaded from the Gene Expression Omnibus (GEO) (https://www.ncbi.nlm.nih.gov/geo/query/acc.cgi?acc=GSE74123). The preparation of samples was described in a previous study[22]. Briefly, fresh CE monolayers with DM were prospectively collected from patients with symptomatic late-onset FECD (clinical stages II to IV) during endothelial keratoplasty (CE transplantation). The RNA of samples was extracted. Eight RNA samples were selected for microarray expression analysis: four FECD (age 75.5 ± 4.0; female: male ratio = 3:1) and four normal control samples (age 52.8 ± 18.8; female: male ratio = 0:4). An Affymetrix Human Gene 1.0 ST Array was used as the platform.

### Microarray data processing and DEGs screening

Data normalization was performed using the “Shengxin.ren” tool, a free software for data analysis (http://gap.shengxin.ren). All samples and genes were clustered by MATLAB. Principal component analysis (PCA) was performed using ImageGP (http://www.ehbio.com/ImageGP/index.php/Home/Index/index.html). Pearson’s Correlation Coefficient was calculated to evaluate the degree of linear correlation between the two samples. The expression of the FECD group was compared to the control group. Genes with | log2 (fold change) | > 1 and P value < 0.05 were selected as the DEGs.

### Functional enrichment analysis of DEGs

All DEGs were submitted to the Database for Annotation, Visualization and Integrated Discovery (DAVID, version 6.8; https://david.ncifcrf.gov/). Gene ontology (GO) terms and Kyoto Encyclopedia of Genes and Genomes (KEGG) pathways were screened with P value <0.05.

### Protein-protein interaction (PPI) network

Protein-protein interaction (PPI) analysis is necessary to illustrate the molecular mechanisms. In this study, the Search Tool for the Retrieval of Interacting Genes (STRING; http://string-db.org/) database was used to construct a PPI network. We picked the genes that appeared more than 10 times in significantly different KEGG pathways. These genes were submitted to STRING. An interaction score of 0.4 was defined as the screened threshold. The important, significant pathways were labeled on the nodes.

## Results

### Preliminary analysis of dataset GSE74123

**Fig 1:**
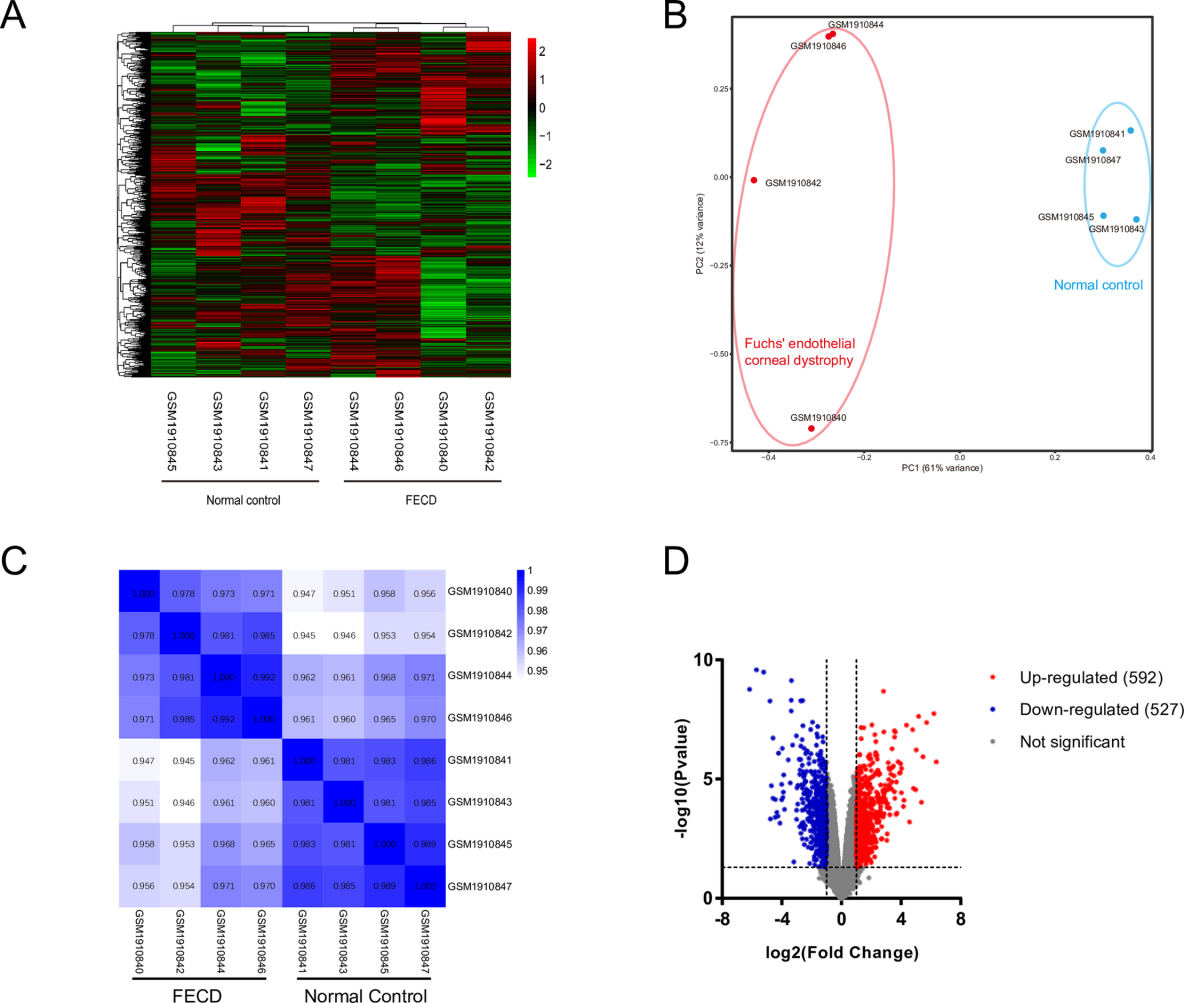
The summary of the dataset GSE74123. (A) The cluster heatmap of GSE74123. (B) The PCA of GSE74123 showed that significant differences between the two groups of samples. (C) Pearson heatmap showed the correlation between the two groups of samples. There was a high correlation within each group of samples. (D) The volcano plot showed the distribution of all genes by fold change and P value. The expression of FECD group was compared with control group. Genes with |log2 (fold change) | > 1 and P value < 0.05 were selected as the DEGs.

GSE74123 was downloaded from the GEO database. After normalization, cluster analysis of the samples and genes was performed. Fig 1A shows significant differences between the FECD group and normal control group. PCA is a statistical method that can analyze the major influencing factors from multiple sources. We performed a PCA analysis of the normalized relative gene expression to show the grouping information for both groups. Similarly, significant differences between the two groups of samples were also found in PCA analysis. Fig 1B shows that the FECD group and control group were clustered into two separate regions, indicating that they had good biological reproducibility. A Pearson’s heatmap (Fig 1C) shows the correlation between the two groups of samples. There was a high correlation within each group of samples, but the control group was higher. A good biological replicate proved that the project's biological experiments can be repeated with little variation.

The expression of the FECD group was compared with the control group. Genes with | log2 (fold change) | > 1 and P value < 0.05 were selected as the DEGs. There were 592 DEGs up-regulated and 527 DEGs that were down-regulated. The volcano plot shows the distribution of all genes in terms of fold change and P value. Red dots represent up-regulated genes, and blue dots represent down-regulated genes (Fig 1D).

These results demonstrated that the FECD group and control group had good biological repeatability. The gene expression of samples in the two groups were significantly different, and thus in-depth bioinformatic analysis could be performed.

### GO enrichment analysis

**Fig 2:**
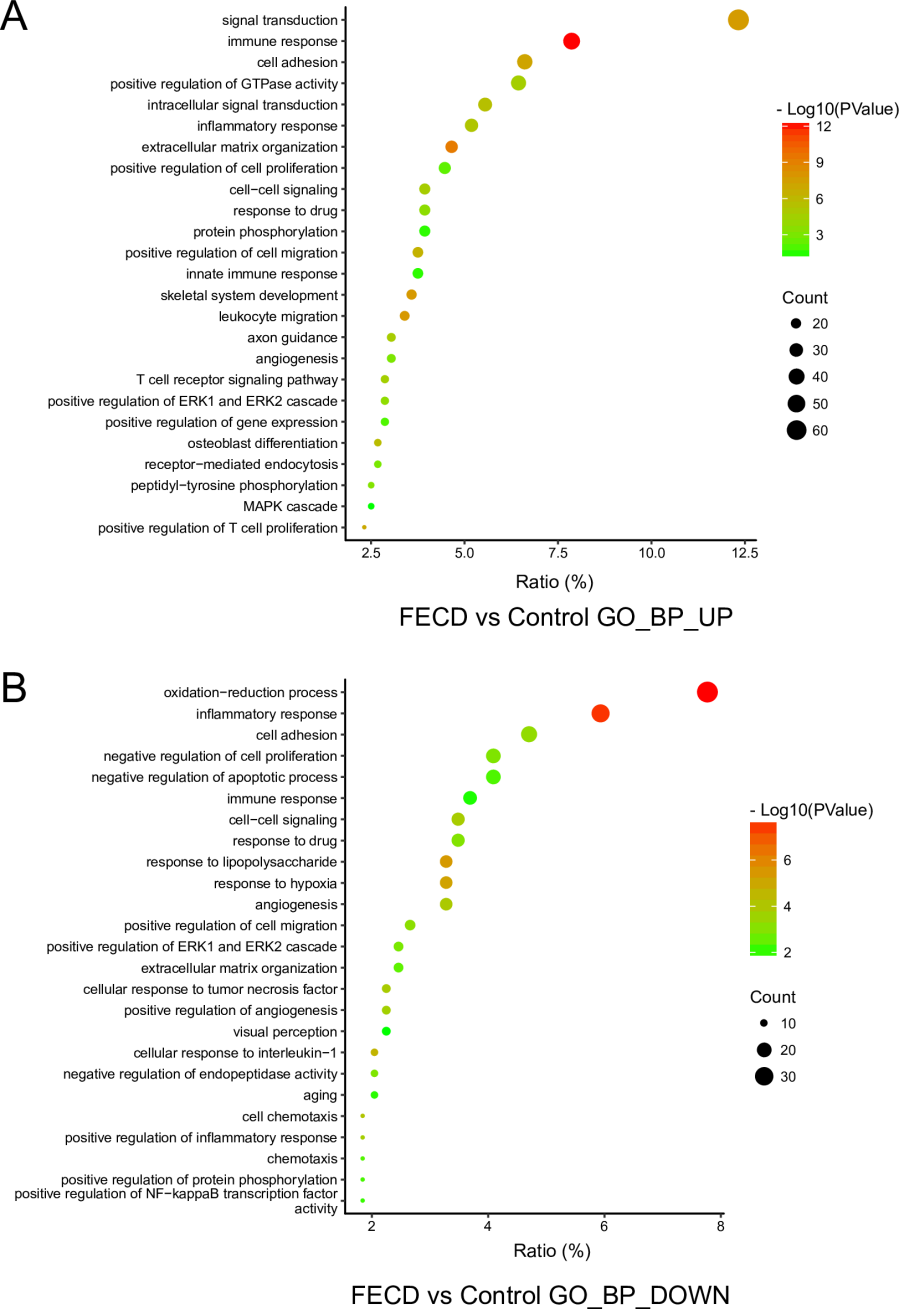
The biological process (BP) in GO enrichment of DEGs. (A) The top 25 significantly up-regulated BP GO terms in FECD group. (B) The top 25 significantly down-regulated BP GO terms in FECD group.

GO terms consist of Biological Process (BP), Cellular Component (CC) and Molecular Function (MF). The up-regulated DEGs and down-regulated DEGs were submitted to DAVID to analyze GO enrichment. The entire list is shown in S1 Table. We picked the top 25 significantly up-regulated BP GO terms (Fig 2A), including immune response, extracellular matrix organization, positive regulation of cell proliferation, and leukocyte migration. The Top 25 significantly down-regulated BP GO terms were also selected (Fig 2B), including oxidation-reduction process, negative regulation of cell proliferation, negative regulation of apoptotic processes, and visual perception. Notably, the second top GO term of up-regulated BP is immune response, and the top GO term of down-regulated BP is oxidation-reduction process. The Top 25 significantly CC and MF GO terms are shown in S1 Fig. The top GO terms of up-regulated CC and MF are plasma membrane and calcium ion binding, respectively. The top GO terms of down-regulated CC and MF are plasma membrane and receptor binding respectively.

### Expression changes of cell senescence, ECM, EMT and immune response related genes

**Fig 3:**
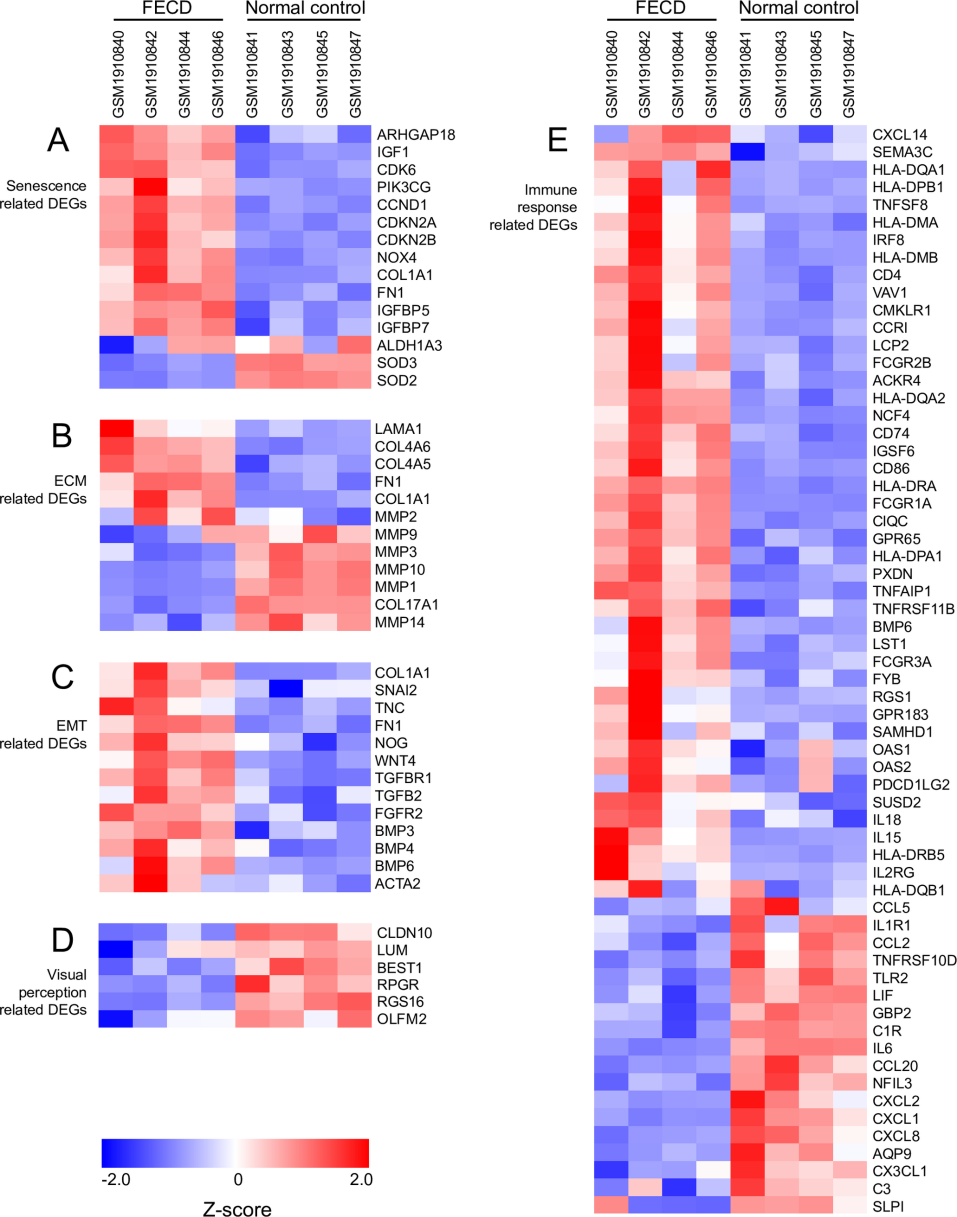
Heatmaps of four major types of genes. (A) Senescence, (B) ECM, (C) EMT, (D) visual perception and (E) immune response related DEGs were picked and normalized by Z-score. Red represents up-regulated expression. Blue represents up-regulated expression.

According to the results of DEGs and GO enrichment, five major types of genes were selected for analysis. First, 15 senescence-related DEGs were selected, based on the study of Matthaei et al.[23]. Compared to the control, genes in FECD related to cell senescence, such as *CDKN2A* and *CDKN2B*, were up-regulated. Oxidase *NOX4* and proliferation genes *CCND1*, *CDK6* were also up-regulated. Antioxidase *SOD2*, *SOD3* were down-regulated (Fig 3A).

Furthermore, the extracellular matrix- (ECM) related genes were up-regulated, and matrix metalloproteinases (MMPs) related genes were down-regulated (Fig 3B). Additionally, epithelial-mesenchymal transition- (EMT) related genes were all up-regulated, especially the key EMT markers *COL1A1*, *SNAI2*, *TNC*, *FN1* and *ACTA2* (Fig 3C). The visual perception-related genes, such as *CLDN10* and *LUM*, were down-regulated (Fig 3D). In addition, in the immune response-related genes, human leukocyte antigen (HLA) genes were up-regulated. *CD4*, *CD74* and *CD86* were up-regulated. Interleukin *IL15* and *IL18* were up-regulated, while *IL6* was down-regulated. Inflammatory chemokines (*CCL2*, *CCL20*, *CXCL1*, *CXCL2*, *CXCL8*) were down-regulated (Fig 3E).

### KEGG signaling pathway analysis

**Fig 4:**
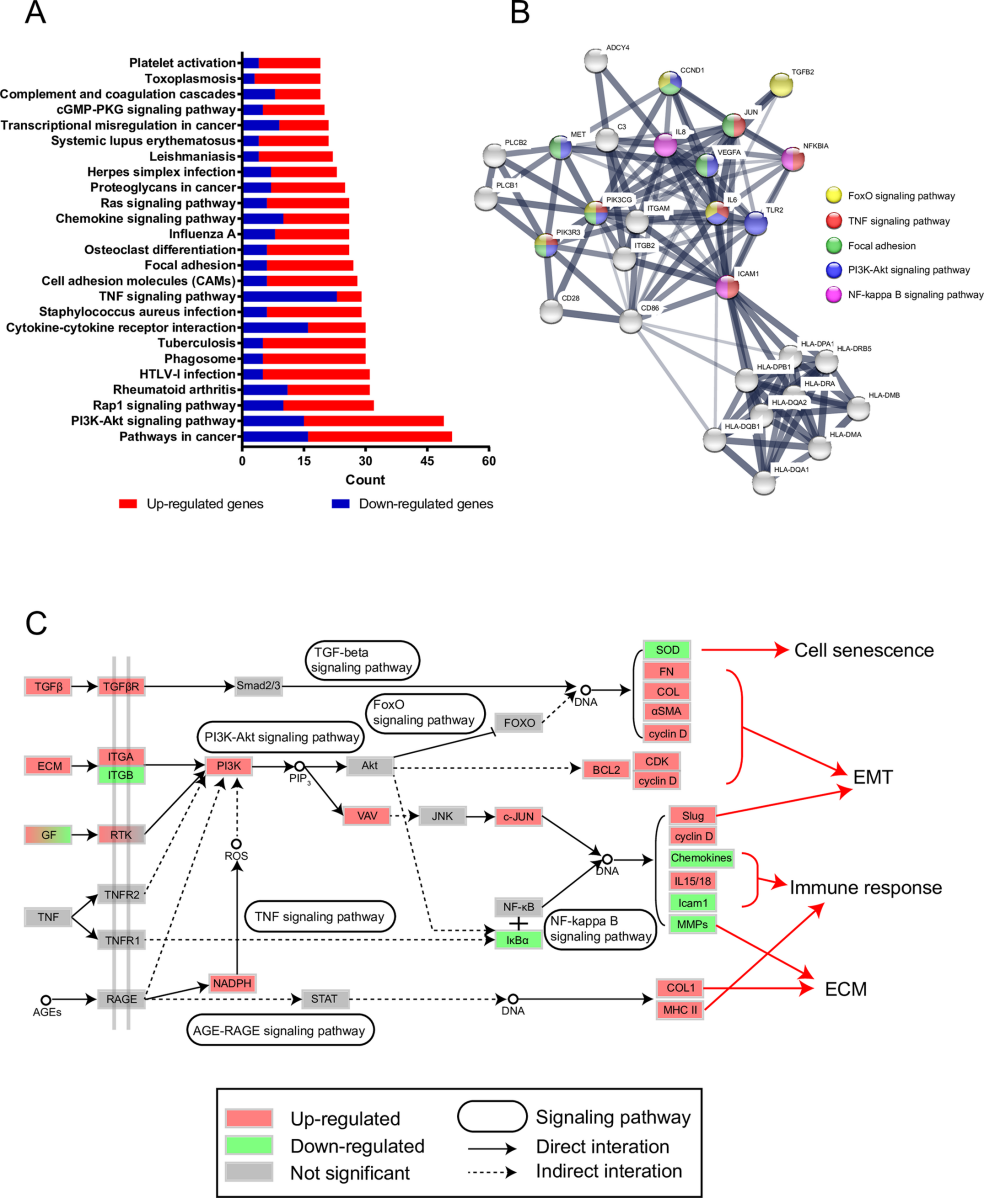
Signaling pathway analysis in symptomatic late-onset FECD. (A) Top 25 significantly different pathways were selected according to DEGs in FECD. (B) PPI of genes which appeared more than 10 times in significantly different pathways. Some important pathways in this study were labeled on it. (C) Signaling pathway network was plotted according to KEGG database.

Various genes coordinate with each other to exercise their biological functions. The most important biochemical metabolic pathways and signal transduction pathways involved in differentially expressed genes can be identified using KEGG significant enrichment. In this study, we submitted all DEGs to DAVID to analyze the KEGG signaling pathways. The entire list is shown in S2 Table. The top 25 significantly different pathways were selected (Fig 4A), including pathways in cancer, the PI3K-Akt signaling pathway, Rap1 signaling pathway, TNF signaling pathway, focal adhesion, and some disease pathways such as rheumatoid arthritis, tuberculosis, Influenza A, and Toxoplasmosis. Then, we listed the genes of all significantly different pathways and counted genes that appeared more than ten times. We plotted PPI with these genes and labeled some important pathways (Fig 4B). It is obvious that PI3K play a key role in the molecular mechanism.

According to the above results, we plotted the possible signaling pathway network (Fig 4C). Up-regulated TGF-β activated its receptor, resulting in increased signal in the TGF-β signaling pathway. EMT-related proteins such as fibronectin, collagen and α-SMA were up-regulated, while cyclin D was also up-regulated. The increase in integrin, growth factor (GF) receptor, TNF receptor and reactive oxygen species (ROS) directly or indirectly activated PI3K protein, which increased the production of phosphatidylinositol (3,4,5)-trisphosphate (PIP_3_), activated the PI3K-Akt pathway and indirectly affected the FOXO signaling pathway, resulting in the down-regulation of SOD2 and SOD3. Up-regulation of PIP_3_ activated Vav and indirectly activated the transcription factor c-Jun. Through the TNF signaling pathway, the expression of IκBα was down-regulated so that the transcription factor NF-κB can enter the nucleus. c-Jun and NF-κB caused up-regulation of Slug, cyclin D, IL15, IL18, and down-regulation of chemokines, ICAM1, and MMPs. The increase in advanced glycation end products (AGEs) led to activation of the AGE-RAGE signaling pathway and up-regulation of collagen I and the major histocompatibility complex (MHC) II.

## Discussion

FECD usually seriously affects binocular vision. It may cause blurred vision or even blindness[24, 25]. Previous studies have found that changes in cell function, such as ECM deposition[26], oxidative stress[27], apoptosis[9] and EMT[26, 28], play a key role in the formation of FECD. However, FECD pathogenesis and the molecular mechanism are incomplete[24–26, 29, 30]. De Roo et al. [22] performed MEA and IPA, listed differentially expressed genes and plotted gene network comprising MHC class II molecules. In this study, we used bioinformatic analysis to select DEGs between FECD and the normal control in GSE74123. The pathological molecular mechanism of FECD is obtained from GO terms and KEGG pathway analysis. We try to explore the molecular mechanisms for the EMT and fibrocyte differentiation in the corneal endothelial layer of symptomatic late-onset FECD.

The main function of CECs is to maintain the transparency of the cornea through the selective barrier and active pump functions. In this study, for symptomatic late-onset FECD, the expression of *CLDN10* (claudin10) was significantly down-regulated in FECD (Fig 3D). This indicates that both the CEC barrier and pump functions are damaged in symptomatic late-onset FECD. Members of the Claudin family play a key role in maintaining tight junctions where located in various types of epithelial cells and endothelial cells[31–33]. Tight junctions form biological barriers with ion selective permeability. CECs positively express claudin-10 and negatively express claudin-14. This protein expression combination can be used to identify CECs[34]. Under normal circumstances, sodium ions enter anterior chamber through tight junctions to enable corneal endothelium pumps to work properly. Claudin-10 plays a key role in sodium ion transport of CEC tight junctions. The down-regulated expression of claudin-10 might prevent sodium ion transport through tight junctions[34].

Furthermore, in the present study, the expression of immune response-related genes was up-regulated. HLA genes were up-regulated. *CD4*, *CD74* and *CD86* were up-regulated. Interleukin *IL15* and *IL18* were up-regulated, while *IL6*, *CXCL1*, *CXCL2*, *CXCL8*, *CX3CL1*, *LIF*, *C3*, *CCL2*, *C1R*, *TLR2*, *GBP2* and *SLPI* were down-regulated (Fig 3E). CD74 is involved in the formation and transportation of MHC class II (HLA) molecules[35]. CD86 is a protein expressed on antigen-presenting cells that provides costimulatory signals necessary for T cell activation and survival[36]. IL15 regulates the activity and proliferation of T cells and NK cells[37]. After stimulation with IL-18, NK cells and certain T cells release another important cytokine—interferon-γ (IFN-γ) or type II interferon—that plays an important role in activating macrophages or other cells[38]. The up-regulation of the above gene expression shows a marked inflammatory and immune response in corneal endothelium and DM in symptomatic late-onset FECD. Meaningfully, here we showed the down-regulated genes of *IL6*, *TLR*, *CXCL1*, *CXCL2*, *CXCL8* in symptomatic late-onset FECD. Previous reports showed that IL-6 skewed the differentiation of dendritic cells into macrophages[39, 40]. IL-6 secretion by dendritic cells followed TLR activation. IL-6 has been shown to inhibit NF-κB activity in dendritic cells[41], suggesting that IL-6 may influence their maturation or trafficking. Normally, IκBα blocks the ability of NF-κB transcription factors to bind to DNA[42]. In this study, IκBα was expressed less in FECD, leading to increased NF-κB activity, further causing changes in the expression of EMT, MMPs and immune-related genes. Therefore, we hypothesize that down-regulated IL-6, enhanced NF-κB activity and a suite of orchestrated chemokine responses induce fibrocyte differentiation from monocytes to dendritic cell maturation in the corneal endothelial layer of symptomatic late-onset FECD.

The production of reactive oxygen species (ROS) may aggravate many inflammatory diseases. There are lots of ROS produced by polymorphonuclear neutrophils (PMNs) in inflammation. While ROS cause vascular endothelial dysfunction and tissue damage [43]. In many kinds of cells, nicotinamide adenine dinucleotide phosphate (NADPH) oxidase leads to the production of large amounts of ROS, especially in phagocytes and vascular endothelial cells[44]. Superoxide dismutase (SOD) not only eliminates the effects of ROS, but it is also an important mechanism for cell anti-apoptosis[45]. In the present study, *NOX4* (NADPH oxidase 4) was significantly up-regulated, while *SOD2* and *SOD3* were significantly down-regulated (Fig 3A). This demonstrated a significant increase in ROS activity in the corneal endothelium of symptomatic late-onset FECD. ROS can induce cell senescence and death. In some cell lines, sublethal amounts of ROS lead to cell cycle arrest and senescence-related changes[46]. In this study, the senescence-related markers *CDKN2A* and *CDKN2B* were significantly up-regulated (Fig 3A), demonstrating the senescence of CECs in symptomatic late-onset FECD. Due to the senescence and death of some CECs, other CECs migrate or alter their shape to maintain the integrity of the corneal endothelial barrier. Corneal endothelial pump function will show a compensatory increase to maintain the corneal deturgescent state[24, 25]. However, as time goes on, the senescence and death of CECs increases, and corneal endothelial barrier function is gradually lost.

Inflammation and ROS can further cause EMT[47]. Befitting EMT is beneficial for wound healing, but excessive EMT causes corneal endothelial fibrosis and the loss of barrier and pump function. Increased EMT leads cells to accumulate more ECM[48]. In this study, for symptomatic late-onset FECD, *COL1A1*, *SNAI2*, *FN1* and *ACTA2* were up-regulated. The accumulation of ECM is the main reason for the thickening of DM and is also a factor in the formation of guttae[29]. The number of guttae is inversely proportional to the corneal endothelial cell density, as the coalescence of guttae is accompanied by a continual loss of CECs[24]. It also results in the loss of corneal endothelial barrier function and further exacerbates corneal edema, forming a vicious cycle.

In this study, we also found that visual perception-related gene expression levels were significantly down-regulated in FECD (Fig 3D). *LUM* (lumican) is a major keratan sulfate proteoglycan of the cornea. It can be combined with collagen fibers, regulating their spatial arrangement[49]. The down regulation of lumican may be related to the disordered arrangement of DM collagen layers in FECD. Interestingly, some retina-related genes (*BEST1*, *RPGR*, *RGS16*, *OLFM2*) were remarkably down-regulated in the present study. Mutations in the *BEST1* (bestrophin 1) gene are causally associated with as many as five clinically distinct retinal degenerative diseases[50]. The Retinitis Pigmentosa GTPase Regulator (*RPGR*) is located in the photoreceptor connecting cilia. It interacts with a wide variety of ciliary proteins[51]. Snow et al. found that *RGS16* (Regulator of G-protein signaling 16) was abundantly expressed in retina, with lower levels of expression in most of the other tissues examined[52]. Sultana et al revealed that olfactomedin 2 (*OLFM2*) may play an important role in the course of retinal and eye development[53]. However, the function of these genes in FECD is currently unknown.

**Fig 5:**
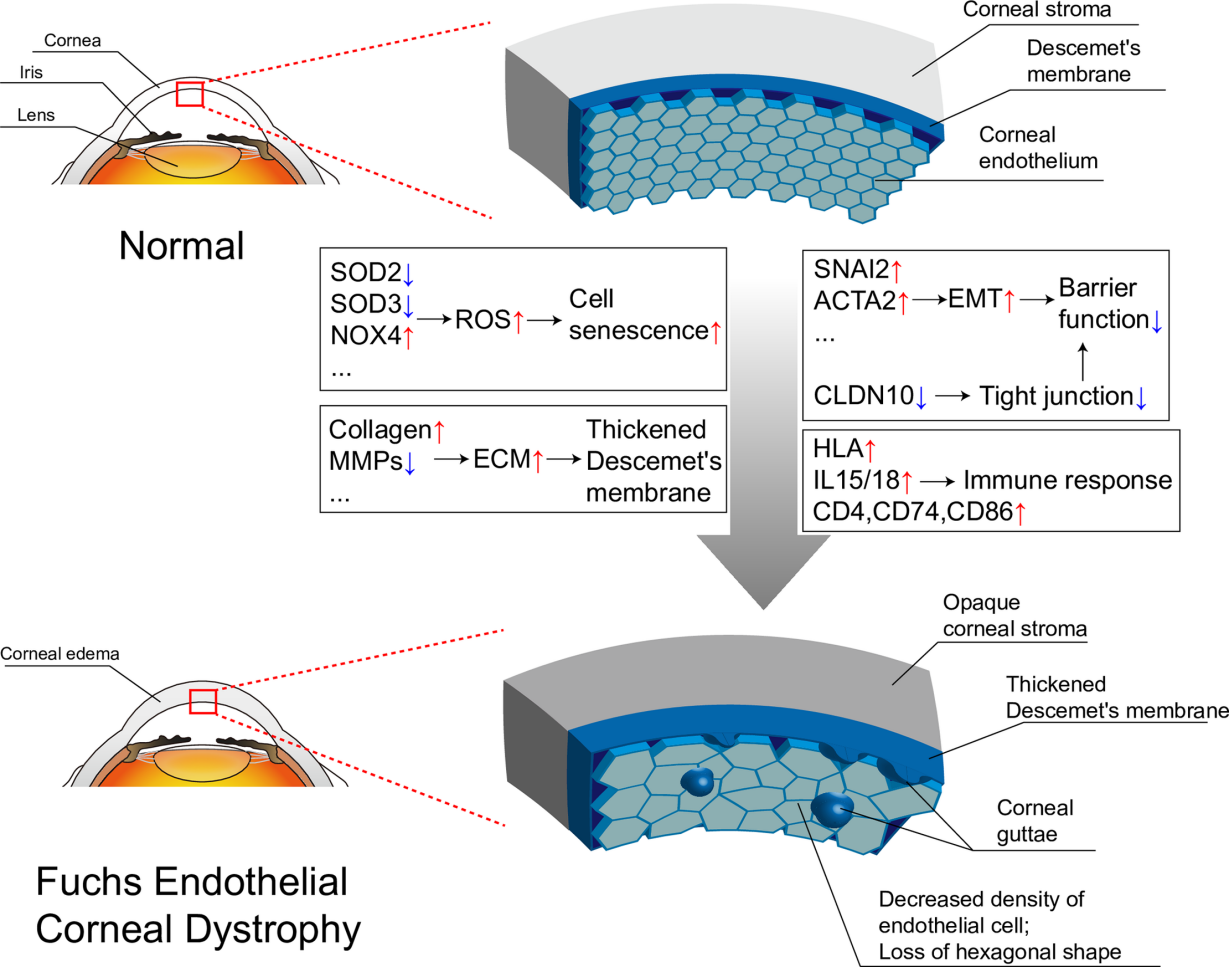
The schematic diagram of possible pathological and molecular mechanisms of symptomatic late-onset FECD. In the symptomatic late-onset of FECD, corneal stroma is cloudy. DM becomes thick. Corneal guttae appear. The density of CECs is decreased. CECs lose their hexagonal shape. The reason for these phenomena is that the expression of cell senescence, EMT, ECM and immune response related genes changes.

In summary, we picked the DEGs of FECD in GSE74123 using bioinformatics analysis. The FECD phenotype and related gene expression levels were related using GO enrichment and KEGG pathway results. We not only confirmed MHC class II type antigen presentation, inflammation, EMT, and ECM production found in previous study, but also drew some new conclusions such as cell senescence, downregulation of *CLDN10* and visual perception related genes. In addition, GO terms, KEGG pathways and PPI network were performed. It was inferred that FECD was related to the PI3K-Akt, TGF-β and NFκB signaling pathway. Down-regulated IL-6, enhanced NF-κB activity and a suite of orchestrated chemokine responses induce fibrocyte differentiation from monocyte to dendritic cell maturation. PI3K plays a key role in the molecular mechanism. The original authors' subsequent research also confirmed the role of NFκB signaling pathway in FECD[11]. The possible pathological and molecular mechanisms of symptomatic late-onset FECD were preliminarily analyzed and diagrammatized (Fig 5). Therefore, for FECD nonsurgical therapy, we might consider therapeutic strategies on anti-inflammatory, anti-ROS and anti-EMT activities, as well as inhibition of NF-κB activity and PI3K activity. However, these conclusions are merely hypotheses, suggested by the results of gene expression analysis, which need further biologically verification in the future.

## Supporting information

S1 Fig The Cellular Component (CC) and Molecular Function (MF) in GO enrichment of DEGs. The top 25 significantly up-regulated CC (A) and MF (B) GO terms in FECD group. The top 25 significantly down-regulated CC (C) and MF (D) GO terms in FECD group.
(PDF)

S1 Table The whole list of GO enrichment. (XLSX)

S2 Table The whole list of KEGG signaling pathway. (XLSX)
