# Supplementary figures and images for: Pathological molecular mechanism of symptomatic late-onset Fuchs endothelial corneal dystrophy by bioinformatic analysis
Source: PLoS One. 2018 May 22;13(5):e0197750. doi: 10.1371/journal.pone.0197750 (PMC5963778; doi:10.1371/journal.pone.0197750)

Figure S1

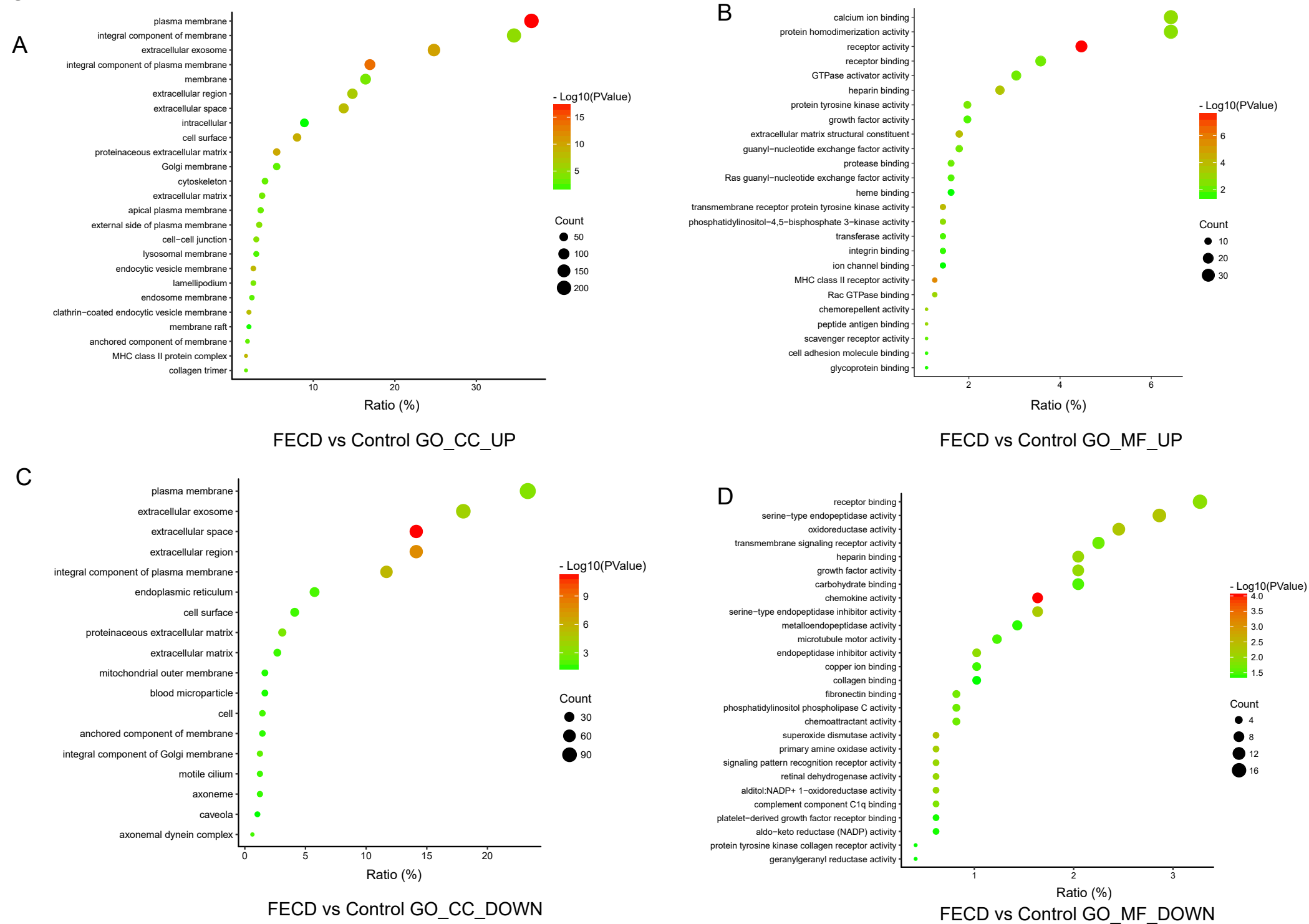

Supplement: S1 Fig — The top 25 significantly up-regulated CC (A) and MF (B) GO terms in FECD group. The top 25 significantly down-regulated CC (C) and MF (D) GO terms in FECD group. (PDF) [file pone.0197750.s001.pdf]
